# Ameliorative effects of silymarin on aflatoxin B1 toxicity in weaned rabbits: impact on growth, blood profile, and oxidative stress

**DOI:** 10.1038/s41598-024-70623-z

**Published:** 2024-09-17

**Authors:** Tharwat A. Imbabi, Abdelkarim I. El‐Sayed, Mohamed H. El-Habbak, Mohammed A. Nasr, Eman H. Halawa

**Affiliations:** 1 Department of Animal Production, Faculty of Agriculture, Benha University, Benha, Egypt; 2 Department of Plant Pathology, Faculty of Agriculture, Benha University, Benha, Egypt; 3 Department of Animal Wealth Development, Faculty of Veterinary Medicine, Zagazig University, Zagazig, Egypt

**Keywords:** Aflatoxin, Silymarin, Growth performance, Rabbit, Physiology, Zoology, Animal physiology

## Abstract

Natural plant extracts offer numerous health benefits for rabbits, including improved feed utilization, antimycotic and antiaflatoxigenic effect, antioxidants, immunological modulation, and growth performance. The aim of the current study was to investigate the effects of silymarin on the performance, hemato-biochemical indices, antioxidants, and villus morphology. A total of 45 Moshtohor 4 weeks old weaned male rabbits were randomly allocated into three groups (15 rabbit/each) each group with 5 replicates. The first group served as the control group feed on an infected diet by aflatoxin B1 (AFB1) 0.02 mg/kg BW, while the second and third groups received an infected diet by AFB1 (0.02 mg/kg BW) and was treated with Silymarin 20 mg/kg BW/day or 30 mg/kg BW/day, respectively. Regarding the growth performance, silymarin supplementation significantly improved the final body weight compared with the control group. Physiologically, silymarin induced high level of dose-dependent total red blood cell count, hematocrit, eosinophils, high-density lipoprotein cholesterol, superoxid dismutase, catalase activity, total antioxidant capacityand intestinal villi width and length. Moreover, silymarin significantly restricted oxidative stress indicators, malondialdehyde, Alanine aminotransferase, aspartate aminotransferase, total cholesterol, triglyceridein rabbits treated with (AFB1). In conclusion, silymarin supplementation to AFB1 contaminated rabbit diet may mitigate the negative effect of AFB1 on the rabbit performance and health status and increase growth performance, average daily gain, immunological modulation and antioxidants and provide a theoretical basis for the application of silymarin in livestock production.

## Introduction

Mycotoxins in cereal products based on the chemical and physical character of the product, storage, processing, rainfall, temperature, humidity and insects^1–3^. Aflatoxins are secreted by various species of fungus Aspergillus, such as A. parasiticus, A. numius, A. pseudotamarii, and A. flavus. Aflatoxin B1 is considered the most crucial and frequently observed mycotoxins due to its high toxicity^4^. It causes oxidative damage through intense production of reactive oxygen species (ROS) that change proteins, DNA and lipids of cellular contents^5^.

Rabbits provide an outstanding source of protein for human consumption. Therefore, many researchers are trying to improve rabbit meat production by selecting high growth rate rabbits with better carcass and meat quality^6–8^. Rabbits are one of the most susceptible animals to toxins such as aflatoxins^9^. Mycotoxicosis in rabbits may lead to considerable reductions in feed intake (up to 60%), delaying growth and resulting in poorer performance and economic loss.

Dietary additives are ingredients used in animal nutrition to improve the feed, nourishment, health and performance of animals^10^. Furthermore, feed additives may not be placed on the market unless a scientific review demonstrates that the additive has no negative effects on human or animal health or the environment. Feed additives are widely used in animal feed for a variety of purposes, including antitoxins, anticoccidial medicines and growth boosters^8,10^. ROS generation is regarded as a pathobiochemical mechanism implicated in the onset or progression of a variety of diseases, including atherosclerosis, ischemic heart disease, diabetes, and the initiation of carcinogenesis or liver ailments^11^.

Phytotherapy is a herbal medicine that is used instead of antibiotic medicine^12^. Silymarin (flavonolignan) is derived from "milk thistle seeds" (*Silybum marianum*). It comprises of flavonoids (silidianin, silychristin, isosilibin, silidianin, silichristin and silybin) that showed antioxidant, anti-inflammatory, anti-fibrotic, anti-lipid peroxidative, immunological stimulant, hepatic cell stabilizing effects and treat cirrhosis^13–15^. Silymarin induces hepatic cells synthesis of ribosomal RNA to encourage protein production^16^ and stops the tumor necrosis factor (TNF)-α production from isolated neurons of Kupffer and the perfused rat liver^17^.

Activation of AFB1 in human and rat liver is a complex operation dominated by multiple cytochrome P450 enzymes^18,19^. Silymarin is a potent antioxidant with hepatoprotective character that prevents the cytochrome P450 system, beats the free radicals and affects the enzymatic systems related to glutathione and superoxide dismutase, consequently inhibits AFB1 activation and elicited changes in liver^20–22^. Therefore, the current study aimed to evaluate the influence of silymarin as an anti-aflatoxin on growth, biochemical performance, and antioxidant markers in rabbits.

## Materials and methods

### Chromatographic condition for AFB1 determination

**Fig. 1:**
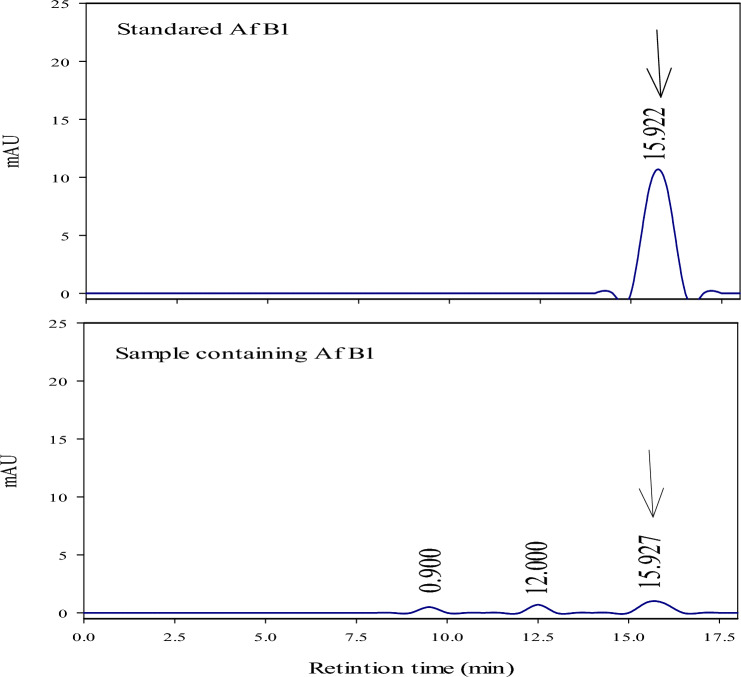
Chromatographic condition for AFB1 determination using HPLC for aflatoxin B1 and standard.

High Performance Liquid Chromatography (HPLC) was used to estimate AFB1 in the feed compared to the standard^23^. The HPLC‐ chromatogram of AFB1 and standard are presented in Fig. 1. The operation conditions were Column packing: Use ODS gel (particle size 3–5 μm) 4.6 mm in inner diameter, 150 mm or 250 mm in length, temperature: 40 °C Mobile phase: Acetonitrile/ methanol/ water (1: 3: 6). Flow rate: 1.0 mL/min, wavelength: Were 365 nm for excitation and 450 nm for emission. AFB1 Standard: Catalog Number: A6636 Potency: ≥ 98% Company: Sigma- Aldrich.

### Animals, experimental design, and environmental data

This study was performed at Faculty of Agriculture, Benha University, Egypt, after the approval of Local Experimental Animal Care Committee (REC-FOABU.16000/3) with confirming that all procedures were done in accordance with the relevant guidelines and regulations. The animals were kept under the standard operating procedures of the University of Benha. The study is reported in accordance with ARRIVE guidelines.

A total of 45 Moshtohor four weeks old weaned male rabbits (developed from crossing between V-line and Gabali)^24^ with an average body weight of 500 ± 5 g were randomly allocated into three treatments, each of which replicated five times with three animals per replicate in cages (45 × 55 × 30 cm) to the end of the experiment (8 weeks). The first group (AF) served as the control group was fed on diet infected with 0.02 mg aflatoxin B1/kg BW, while the second and third groups received diet infected with 0.02 mg aflatoxin B1/kg BW and treated with Silymarin 20 mg/kg BW/day (second group) or 30 mg/kg BW/day (third group). Supplementations were given orally daily during the experiment period. During the experimental period, rabbits were fed the same standard iso-caloric/iso-nitrogenic diet. The basal diet composition and calculated analysis were performed in accordance with the Nutritional Research Council Nutrient Requirements of Rabbits^25^ as shown in Table 1.

**Table 1:** Composition and calculated analyses (g/kg) of the experimental basal diet.

Ingredients	Content
Alfalfa hay	350
Yellow corn	200
Soybean meal	96
Wheat bran	300
Corn Stover	30
Di-calcium phosphate	12.5
l-Lysine HCl	2.1
DL-Methionine	2
Sodium chloride	5
Vitamin/mineral premix*	1.5
Total	1000
Calculated analysis (g/ kg on dry matter basis)	
Digestible energy (MJ/kg)	11.6
Crude protein	179
Crude fiber	125
Crude fat	32.0
Ca	10.9
Available P	5.9
Methionine	4.2
Lysine	9.0

*Each 1 kg of premix (minerals and vitamins mixture) contains vit. A, 20,000 IU; vit. D3, 15,000 IU; vit. E, 8.33 g; vit. K, 0.33 g; vit. B1, 0.33 g; vit. B2, 1.0 g; vit. B6, 0.33 g; vit. B5, 8.33 g; vit. B12, 1.7 mg; pantothenic acid, 3.33 g; biotin, 33 mg; folic acid, 0.83 g; choline chloride, 200 g.

### Growth performance

Weaned rabbits in each replicate were weighed at 4, 8, 12 weeks of age using a digital scale and the average daily weight gain (ADG (g/weaned rabbit) was calculated. At the end of the experiment, the carcass characteristics were assessed (For more details see^26^). In addition, rabbit *Musculus Longissimus lumborum* (LL) was dissected and the liver was cleaned and frozen at – 80 °C for assessment of meat quality and antioxidant status.

### Meat quality parameters

Right and left *Longissimus lumborum* muscles (LL) were collected to determine the pH, water holding capacity (WHC), drip loss, thawing and cooking loss (samples taken 10 min to reach 75 °C in preheated water bath), Warner–Bratzler Shear Force (WBSF), lightness (L*), redness (a*), yellowness (b*), chroma (C), and Hue angle (h)^27^. Additionally, moisture contents were assessed by AOAC^28^. The keeping quality of rabbits meat was evaluated over 10 days (For more details see^26,29^), then APC was determined and pH was measured ( For more details see^26,30^).

### Hematological and biochemical parameters

At the end of the experimental period, two blood samples were collected. The first sample was collected with anticoagulant (EDTA) to determine hematocrit (HCT), hemoglobin (Hgb), mean corpuscular volume (MCV), mean corpuscular hemoglobin (MCH) and mean corpuscular hemoglobin (MCHC), total red blood cell count (RBC) and total white blood cell count (WBC)^31^. The second sample was collected without anticoagulant, and the serum stored at − 20 °C until the biochemical parameters measured (For more details see^26^). Alanine aminotransferase (ALT), aspartate aminotransferase (AST) were determined Morgenstern et al.^32^. Serum total cholesterol, triglyceride and high-density lipoprotein cholesterol (HDL-C) were spectrophotometrically assessed using commercial kits developed by Pasteur laboratories (Egyptian American Co. for Laboratory Services, Egypt).

### Antioxidant profile

Rabbit's livers were washed with phosphate buffered saline (PBS) solution (pH 7.4 with 0.16 mg/ml heparin) to eliminate any red blood clots. The liver was homogenized in 5 mL of cold PBS per gram of tissue (1:5 dilution). Samples were centrifuged at 4000 rpm at 4 °C for 15 min. Supernatants were collected and stored at − 20 °C until biochemical analysis of superoxid dismutase (SOD)^33^, catalase activity^34^, malondialdehyde (MDA)^35^ and total antioxidant capacity (TAC) levels^36^.

### Quantitative histomorphometric analysis of jejunum segments

Five Segments of the mid-jejunum (3 cm) from each treatment were collected, fixed with formalin for 48 h and paraffin embedded. Two sections (100 μm) from each sample were obtained, stained with hematoxylin for one min., and counterstained with eosin for 10 s. to assess the maximum villus length (measured from above the crypt to the tip of the villus), villus width, and submucosa/muscularis/serosa thickness. All targeted variables were measured with a camera (OLYMPUS; TH4-200; Tokyo, Japan) coupled with computer-assisted digital-image pro plus (IPP) analysis software (Image-Pro Plus 4.5, Media Cybernetics, Silver Spring, MD, USA).

### Statistical analysis

Data analysis was carried out using PROC GLM^37^ and expressed as mean value ± SEM. Differences were considered to be statistically significant at P < 0.05. The difference among treatments were tested using Duncan’s multiple range test at P < 0.05. The static model applied is as follow:

$$ {\text{yij}} = \mu + {\text{Ti}} + {\text{eij}} $$

where: y is the observations, µ = general mean, Ti: effect of treatment (i = 1, 2 and 3), eij: random error.

### Ethical approval

This study was performed at Faculty of Agriculture, Benha University, Egypt, after the approval of Local Experimental Animal Care Committee (REC-FOABU.16000/3) with confirming that all procedures were done in accordance with relevant guidelines and regulations. The animals kept under the standard operating procedures of the University of Benha. The study is reported in accordance with ARRIVE guidelines

## Results

### Growth performance and carcass traits

The results illustrated that adding silymarin increased (p < 0.05) the BW8, BW12, ADG4-8, ADG 8-12and ADG4-12 compared to the AF group Table 2. The growth performance of rabbits supplemented with Silymarin 20 mg (501.7, 1124.0 and 1684.7 g) and 30 mg (499.3, 1110.0 and 1700 g) were better than the AF group from week 4–8, 8–12 and 4–12, respectively (Table 2). There was no significant difference among the experimental groups regarding the relative weights of carcass cut and internal organs except for the head Table 3.

**Table 2:** Impact of dietary silymarin on growth performance of weaned Moshtohor rabbits fed diet infected with aflatoxin.

Growth parameter	AF	AF + Silymarin 20 mg	AF + Silymarin 30 mg	SEM	P Value
Body weight (BW) (g)					
BW4	501.0	501.7	499.3	1.69	0.63
BW8	950.3^b^	1124.0^a^	1110.0^a^	5.67	0.0001
BW12	1343.0^b^	1684.7^a^	1700.6^a^	36.28	0.0040
Average daily gain (ADG)(g/d)					
ADG4-8	16.02^b^	22.23^a^	21.77^a^	0.21	0.0001
ADG8-12	14.04^b^	20.00^a^	21.10^a^	1.26	0.045
ADG4-12	15.13^b^	21.10^a^	21.47^a^	0.64	0.004
FI (g/day)	50.33	50.70	50.93	0.29	0.401
FCR (%)	2.86^a^	2.61^b^	2.06^c^	0.10	0.004

^a,b,c^ Letters indicated significant differences among means within the same row (p < 0.05). AF: Rabbits fed on diet infected with 0.02 mg aflatoxin B1 /kg BW. AF + Silymarin 20 mg: Rabbits received diet infected with 0.02 mg aflatoxin B1 /kg BW and treated with Silymarin 20 mg/kg BW/day. AF + Silymarin 30 mg: Rabbits received diet infected with 0.02 mg aflatoxin B1 /kg BW and treated with Silymarin 30 mg/kg BW/day. BW4: initial body weight at four weeks; BW8: body weight at eight weeks; BW12: final body weight at 12 weeks; ADG8-4: average daily gain from 4 to 8 weeks; ADG8-12: average daily gain from 8 to 12 weeks; ADG4-12: average daily gain from 4 to 12 weeks. FI: feed intake, FCR %: Feed conversion ratio.

**Table 3:** Impact of dietary silymarin on the relative weights of carcass cuts and internal organs of weaned Moshtohor rabbits fed diet infected with aflatoxin.

Parameters	AF	AF + Silymarin 20 mg	AF + Silymarin 30 mg	SEM	P Value
Live body weight(g)	1343.00^b^	1684.67^a^	1700.67^a^	36.28	0.0004
Carcass (%)	50.20	46.06	48.03	2.43	0.26
Head rate (%)	6.29^a^	5.10^b^	5.42^b^	0.36	0.03
Hind legs rate (%)	13.13	12.21	14.23	1.03	0.37
Saddle rate (%)	9.33	10.47	10.59	0.41	0.23
Fore legs rate (%)	9.10	9.43	10.05	0.24	0.32
Thoracical neck rate (%)	11.42	9.45	12.37	1.25	0.15
Liver (%)	2.46	2.30	2.18	0.17	0.21
Kidney (%)	0.77	0.68	0.65	0.05	0.28
Spleen (%)	0.064	0.059	0.045	0.01	0.19
Lung (%)	0.79	0.63	0.66	0.07	0.47
Heart (%)	0.20	0.26	0.21	0.02	0.13

^a,b,c^Letters indicate significant differences among means within the same row (p < 0.05). AF: Rabbits fed on diet infected with 0.02 mg aflatoxin B1 /kg BW. AF + Silymarin 20 mg: Rabbits received diet infected with 0.02 mg aflatoxin B1 /kg BW and treated with Silymarin 20 mg/kg BW/day. AF + Silymarin 30 mg: Rabbits received diet infected with 0.02 mg aflatoxin B1/kg BW and treated with Silymarin 30 mg/kg BW/day. The parameters rate and organ index were calculated as follows: parameters rate or organ index = (organ weight/living weight) × 100%.

### Physical characteristics and microbial abundance of MLD muscle

The current results did not reveal any significant differences regarding WHC, drip loss (48 h), thawing loss, cooking loss, lightness (L*), redness (a*) and yellowness (b*) and Hue angle (h) of *Longissimus Lumborum* muscle. While silymarin supplementation decreased shear force with an increase of moisture content when compared with the AF group (P < 0.05). Chroma was increased in the group provided with silymarin 20 mg (P < 0.05) (Table 4), but the pH values were lower than the AF group (P < 0.05).

**Table 4:** Impact of dietary silymarin on meat quality of weaned Moshtohor rabbits fed diet infected with aflatoxin.

Meat quality	AF	AF + Silymarin 20 mg	AF + Silymarin 30 mg	SEM	P value
pH (24 h)	5.66^b^	6.00^a^	5.85^b^	0.02	0.000
WHC	82.26	84.65	83.51	2.82	0.73
Drip loss (48 h) %	1.23	1.40	1.22	0.19	0.67
Thawing loss	5.65	6.16	9.25	1.37	0.43
Cooking loss %	22.49	17.09	18.56	1.27	0.28
WBSF	6.68^a^	5.44^b^	4.12^c^	0.25	0.0001
L*	53.54	53.39	50.79	0.69	0.24
a*	10.70	11.75	13.85	0.84	0.72
b*	5.56	7.23	5.86	0.19	0.08
C	12.59^b^	12.34^b^	15.15^a^	0.30	0.03
h°	27.71	29.86	24.08	0.84	0.05
Moisture	70.30^b^	72.57^b^	76.89^a^	0.34	0.009

^a,b,c^Letters indicate significant differences among means within the same row (p < 0.05). AF: Rabbits fed on diet infected with 0.02 mg aflatoxin B1 /kg BW. AF + Silymarin 20 mg: Rabbits received diet infected with 0.02 mg aflatoxin B1 /kg BW and treated with Silymarin 20 mg/kg BW/day. AF + Silymarin 30 mg: Rabbits received diet infected with 0.02 mg aflatoxin B1 /kg BW and treated with Silymarin 30 mg/kg BW/day. WHC: Water holding capacity.

*WBSF* Warner–Bratzler Shear Force. L* lightness, a* redness, b* yellowness, *C* chroma, *h°* hue angle.

### Blood hematological and biochemical

The effects of silymarin supplementation on hematological and biochemical variables of blood are summarized in Table 5. There were no statistically significant differences of hematological variables among the treated groups except for RBCS, hematocrit, MCV and eosinophils. Rabbits supplemented with Silymarin 20 mg and 30 mg exhibited highest RBS`s count hemoglobin and platelets than those of the AF group. Meanwhile, the group supplemented with Silymarin 20 mg or 30 mg had a significantly lower hematocrit and eosinophils percentage than the AF group. Generally, weaning rabbits supplemented with Silymarin 20 mg or 30 mg revealed a reduction of the total cholesterol, triglyceride, ALT, AST and creatinine than the AF weaning rabbits. The provision of silymarin 30 mg had a significantly increase in the concentration of HDL. Supplementation of silymarin to the diet infected with aflatoxin led to reduce significantly the liver and kidney function compared with the AF group (Table 5).

**Table 5:** Impact of dietary silymarin on hematological and biochemical variable of blood serum of weaned Moshtohor rabbits fed diet infected with aflatoxin.

Parameters	AF	AF + Silymarin 20 mg	AF + Silymarin 30 mg	SEM	P value
Hematological variable of blood					
Hemoglobin (g/dL)	16.97	17.60	19.00	0.62	0.14
R.B.Cs (10^6^/cmm)	5.65^b^	6.46^a^	7.800^a^	0.21	0.006
HTC (vol%)	42.00^ab^	40.50^b^	47.00^a^	0.90	0.03
MCV (fl)	71.30^a^	58.97^b^	59.60^b^	1.57	0.003
MCH (pg)	29.17	26.10	27.60	1.11	0.15
MCHC (%)	39.83	46.17	42.30	2.13	0.59
Platelets (10^3^/cmm)	653.5	698.0	665.5	63.09	0.77
WBC (10^3^/cmm)	9.20	8.77	10.76	0.73	0.97
Neutrophils %	66.00	69.00	67.50	1.12	0.32
Lymphocytes %	23.30	24.00	23.00	1.26	0.88
Monocytes%	4.58	3.10	5.00	0.62	0.07
Eosinophils %	2.00^a^	0.400^c^	1.40^b^	0.24	0.01
Basophils %	0	0	0	0	0
Biochemical variables of blood serum					
Total Cholesterol (mg/dL)	79.59^a^	65.48^b^	56.11^c^	2.92	0.002
High density lipoprotein (mg/dL)	56.31^a^	42.82^b^	45.78^b^	2.22	0.01
Triglyceride (mg/dL)	26.39^a^	22.67^b^	19.28^c^	0.58	0.0004
Liver function					
AST (U/I)	48.76^a^	32.75^b^	25.29^b^	2.244	0.0008
ALT (U/I)	65.02^a^	57.59^b^	55.12^b^	11.14	0.7707
Kidney function					
Creatinine(mg/dl)	3.42^a^	2.08^b^	2.00^b^	0.094	0.0762

^a,b,c,d^ letters indicate significant differences among means within the same row (p < 0.05). AF: Rabbits fed on diet infected with 0.02 mg aflatoxin B1 /kg BW. AF + Silymarin 20 mg: Rabbits received diet infected with 0.02 mg aflatoxin B1 /kg BW and treated with Silymarin 20 mg/kg BW/day. AF + Silymarin 30 mg: Rabbits received diet infected with 0.02 mg aflatoxin B1 /kg BW and treated with Silymarin 30 mg/kg BW/day.

*RBC* total red blood cell count, *HCT* hematocrit, *M.C.V.* mean corpuscular volume, *M.C.H*. mean corpuscular hemoglobin, *M.C.H.C* mean corpuscular hemoglobin concentration, *WBC* total white blood cell count, *AST* aspartate aminotransferase, *ALT* alanine aminotransferase.

### Antioxidant enzyme activity

Results of antioxidant markers of weaned rabbits as they were influenced by the supplementation are demonstrated in Table 6. Compared with AF group, the levels of SOD, CAT and TAC were increased at week 12 of rabbit provided with silymarin with a reduction of MDA.

**Table 6:** Impact of dietary silymarin on the antioxidant parameter in liver tissue of weaned Moshtohor rabbits fed diet infected with aflatoxin.

Antioxidant parameter	AF	AF + Silymarin 20 mg	AF + Silymarin 30 mg	SEM	P value
MDA (nM/g)	275.42^a^	240.96^b^	223.20^c^	3.240	0.0001
SOD (Ul/µmL)	155.73^c^	173.63^b^	195.31^a^	5.452	0.0001
CAT (u/mg)	3.16^b^	3.85^a^	3.96^a^	0.101	0.0091
TAC (mM/g)	8.56^b^	12.67^a^	13.45^a^	0.229	0.0001

^a,b,C^letters indicate significant differences among means within the same row (p < 0.05). AF: Rabbits fed on diet infected with 0.02 mg aflatoxin B1 /kg BW. AF + Silymarin 20 mg: Rabbits received diet infected with 0.02 mg aflatoxin B1 /kg BW and treated with Silymarin 20 mg/kg BW/day. AF + Silymarin 30 mg: Rabbits received diet infected with 0.02 mg aflatoxin B1 /kg BW and treated with Silymarin 30 mg/kg BW/day.

*MDA* malondialdehyde, *SOD* superoxid dismutase, *CAT* catalase, *TAC* total antioxidant capacity.

### Villus morphology and morphometry

**Fig. 2:**
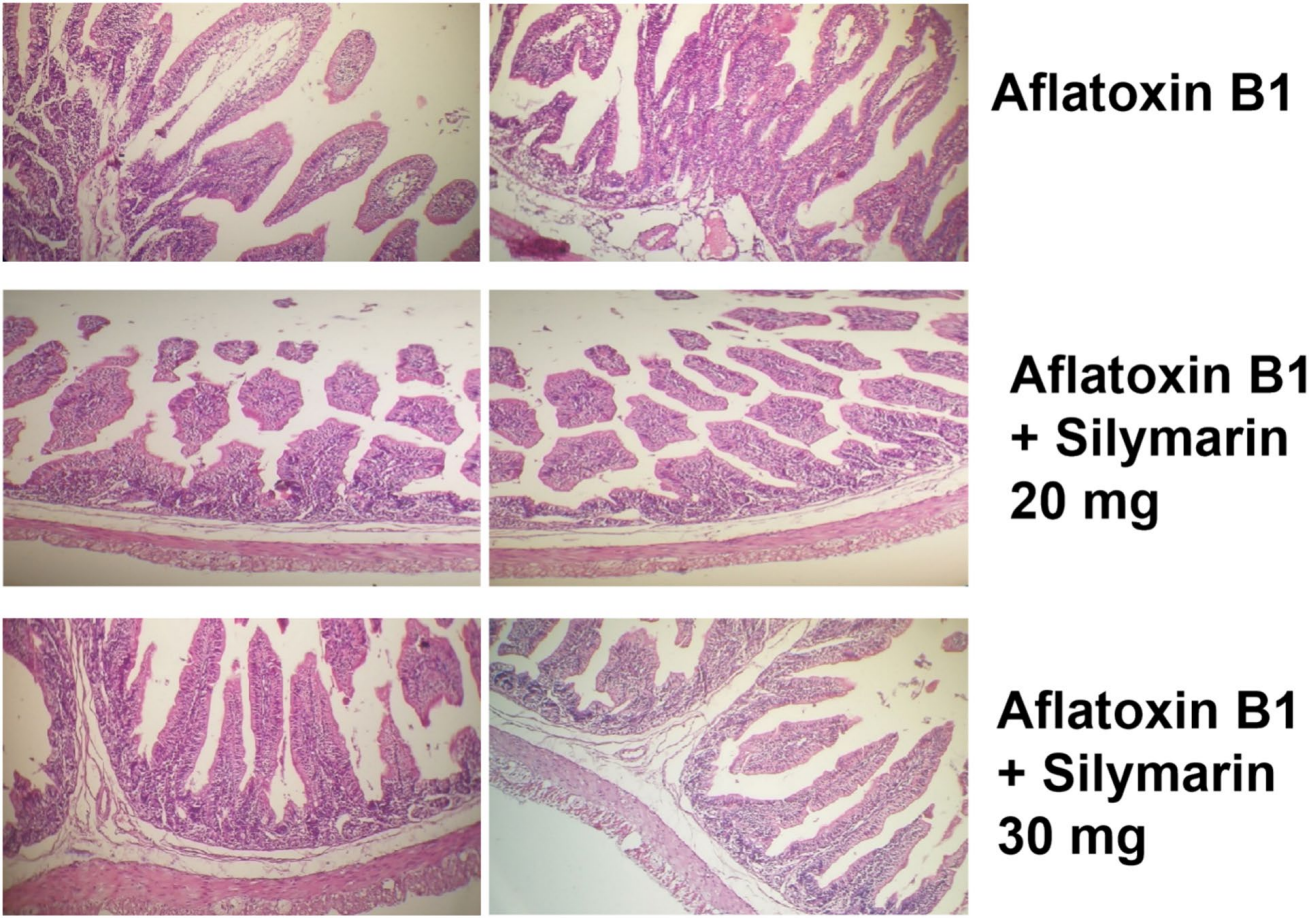
Histological image for the impact of dietary silymarin on villus morphology and morphometry of 12 weeks old rabbits fed diet infected with aflatoxin. Rabbits fed on diet infected with 0.02 mg aflatoxin B1/kg BW. aflatoxin B1 + Silymarin 20 mg: Rabbits received diet infected with 0.02 mg aflatoxin B1/kg BW and treated with Silymarin 20 mg/kg BW/day. aflatoxin B1 + Silymarin 30 mg: Rabbits received diet infected with 0.02 mg aflatoxin B1/kg BW and treated with Silymarin 30 mg/kg BW/day.

Data regarding intestinal morphology of weaned Moshtohor rabbits aged 12 weeks are shown in Table 7 and Fig. 2. Although the NVIS was consistently higher in the additives group, it was not statistically significant. However, the weaned rabbit which received Silymarin 20 mg or 30 mg had a higher villus width and length than those in the AF group. Furthermore, the supplementation of Silymarin 20 mg or 30 mg improved the MTh and G cell at 12 weeks of age rabbit.

**Table 7:** Impact of dietary silymarin on villus morphology and morphometry of 12 weeks old rabbits fed diet infected with aflatoxin.

Parameters	AF	AF + Silymarin 20 mg	AF + Silymarin 30 mg	SEM	P value
NVIS	45.11	55.00	57.00	4.789	0.4016
Villus width	101.00	118.00	129.00	9.695	0.0890
Villus length	351.11^b^	477.00^a^	526.44^a^	19.14	0.0001
MTh	54.00^b^	67.00^ab^	99.00^a^	5.123	0.0079
Gcell	15.56	17.20	19.95	1.424	0.7434

^a,b^Letters indicate significant differences among means within the same row (p < 0.05). AF: Rabbits fed on diet infected with 0.02 mg aflatoxin B1 /kg BW. AF + Silymarin 20 mg: Rabbits received diet infected with 0.02 mg aflatoxin B1 /kg BW and treated with Silymarin 20 mg/kg BW/day. AF + Silymarin 30 mg: Rabbits received diet infected with 0.02 mg aflatoxin B1 /kg BW and treated with Silymarin 30 mg/kg BW/day.

*NVIS* no. of villin section, *MTh* musclaris thickness, *Gcell* goblet lining cells.

## Discussion

Aflatoxin B1 (AFB1) is the most abundant hazard metabolite and the most common feed contaminant in many parts of the world. When more than one mycotoxin present in feed, the toxicity and clinical symptoms in animals are complicated and diverse. The exposure duration (acute or chronic), contamination rate of AFB1 in diet, animal species, age, production status, and toxin co-contamination (synergistic effect) may influence animal’s response to the toxin^38^. Therefore, the current study aimed to evaluate the influence of silymarin as an anti-aflatoxin on growth, biochemical performance, and antioxidant markers in rabbits.

The current results revealed a reduction of the growth performance of rabbits fed diet contaminated with AFB1. These results are in agreement with the results obtained by Farag et al.^39^; Sun et al*.*^40^. The performance deterioration caused by the toxins may be related to several factors such as; (a) decreased feed intake, (b) modification of liver oxygenase enzymes activity that responsible for protein, carbohydrate and lipid metabolism, (c) modification of essential nutrients absorption concurrently with patho-anatomical alterations in vital organs, especially liver^41–43^, (d) toxic influence on the cellular contents via inhibition of protein, RNA and DNA syntheses^44^, (e) decreased digestibility of nitrogen free extract, ether extract and dry matter, (f) anorexia and lipogenesis^45,46^.

Supplementation of 20 or 30 mg/kg silymarin in the current study, significantly improved the growth, live body weight and average daily gain compared with AF (control groups). These growth-promoting benefits could be owned to silymarin's hepato-protective and immune-boosting properties. Indeed, it has been proposed that the presence of functional bioactive ingredients in plant extracts, such as flavonoids and phenolic compounds, could improve feed digestibility and nutrient bioavailability. Thereby increasing feed utilization and promoting higher protein synthesis^47,48^. The growth improvement may be associated with the availability of bioactive functional phytochemicals that might improve feeding intake, feed efficiency, and protein retention. Furthermore, silymarin content may increase protein synthesis via the enzymatic system^49^. Banaee et al*.*^49^ reported that supplementation with S. marianum changes the expression of the growth hormone gene that participates in muscle fibers creation. These results are comparable with Hasheminejad et al.^50^ who reported that silymarin reduced the toxic effects of AFB1 and the metabolic needs of the digestive tract. Consuming 0.5% S. marianum reduces the pathogenic microorganisms in ileum^51^.

The current findings regarding the carcass traits revealed numerically improvement by adding silymarin but the differences were non-significant. These results are comparable to others that stated the weights and relative weights of internal organs (spleen, brain, liver and kidney) did not reveal differences between NZW rabbits orally treated with AFB1 and the control group^39,52^. There were no significant changes in the rheological qualities of rabbit meat when silymarin was added by 20 and 30 mg. This could be because silymarin has powerful antioxidant effects^53^ by (a) direct free radical scavenging, blocking specific enzymes responsible for free radical production (b) by maintaining the integrity of the electron-transport chain of mitochondria under stress conditions, contributing to the cell's optimal redox status by activating a variety of antioxidant enzymes and non-enzymatic antioxidants. This may be primarily via transcription factors such as nuclear factor erythroid 2-related factor 2 (Nrf2), a protein that regulates the expression of antioxidant proteins that protect against oxidative damage caused by injury and inflammation, and nuclear factor kappa-light-chain enhancer of activated B cells (NF-kB)^54^.

Blood is a connective tissue made up of fluid or plasma sections suspended by elements (erythrocytes, leukocytes, and thrombocytes). Blood connects the body's organs and cells to maintain a continuous cellular environment by circulating through every tissue, giving nutrients, and removing waste materials. The current results explained that there was a significant improvement in RBCs, HCT and Eosinophils due to the treatment with silymarin 20 and 30 mg/kg B.W. compared with the control group. Also, obtained results are in agreement with the finding of Farag et al.^39^ who reported that rabbits fed on contaminated diet with AFB1 showed higher serum TC, TG and LDL levels compared with the other groups. Silymarin administration resulted in decreased total cholesterol, triglyceride, and increased HDL levels, which could be linked to silymarin's antioxidant action^55,56^.

Silymarin is lipophilic and highly binds to plasma membrane components, improving plasma membrane strength and reducing membrane rupture and disintegration^57^. AFB1 can be easily recognized in infected animals' hepatic tissues and it is regarded as one of the most important body organs due to the way it detoxifies or eliminates toxins and foreign materials^58^. The current results revealed that AF reduced the RBCs and WBCs this was comparable to others^59,60^ that might be due to the hazard effect of aflatoxin on liver tissues, which in turn caused impairment of hematopoietic tissue^61^. Moreover, Sun et al*.*^40^ stated that male rabbits fed with high level of AFB1 showed decrease of RBC, compared to bucks fed a low level of AFB1 or the control group. This reduction is represented by the anaemic consequence of aflatoxicosis that could be owned to the reduction of serum iron and modified protein metabolism. The hazard impact of AFB1 on hematology was alleviated by silymarin that were orally intake^62^. Moreover, Abou-Shehema et al*.*^56^ observed that cockerels fed diets provided with 25 g Milk thistle/kg diet (equal to 1 g silymarin /kg diet) revealed significantly improved RBCs and WBCs compared to the control group during the summer season.

The effects of AFB1 on the liver were reflected in blood biochemistry data, particularly ALT, AST, and serum proteins. The serum enzyme activity results are in agreement with Hatipoglu and Keskin^63^, who found that AFB1-intoxicated rats had significantly higher levels of liver enzymes than control rats. Silymarin can impact the metabolism and concentration of lipids in the blood by reducing cholesterol synthesis in the liver and lowering blood cholesterol by limiting its absorption in the gastrointestinal tract^64^. Obtained results agreed with Sobolová et al*.*^65^ who demonstrated that silymarin reduced blood cholesterol and triglycerides and can impact the metabolism and concentration of lipids in the blood by reducing cholesterol synthesis in the liver and lowering blood cholesterol by limiting its absorption in the gastrointestinal tract.

Silymarin protects liver cells from viruses, chemicals, and natural toxins like aflatoxins (fungal toxins). There have been several reports of improved liver function following silymarin prescription (administration)^66^. It can also prevent the peroxidation processes involved in liver lesions produced by toxins and other hazardous chemicals^16^. Creatinine is a chemical byproduct of muscle metabolism, when kidneys are healthy; they filter creatinine and other waste products from the blood. Silymarin supplementation in combination with AFB1 improved creatinine by decreasing levels due to the kidney filtration functions. The present results agreed with Ashrafihelan et al*.*^67^ who reported that oral therapy of silymarin (13 mg/kg BW) resulted in better kidney function. Antioxidant markers were improved in blood of weaned rabbits due to the use of silymarin + AFB1, this improvement is related to the effect of silymarin as antioxidant.

The liver and kidneys are detoxifying organs (mycotoxins’ metabolism). ALT and AST are used as a chief index to assess liver performance^68^ because they are released into bloodstream when the liver is damaged. The high levels of AST and ALT are indicators of liver damage^69^. AF in diet of rabbits elevated the values of ALT and AST in blood as a result of hepatocytes loss of their integrity with destruction of hepatic parenchymal cells. Hassan et al.^70^ and Farag et al.^39^ reported that rabbit fed with diet contaminated with AFB1 showed an increase of liver enzymes which supported the current findings. Moreover, AFB1 caused liver cells cirrhosis and necrosis^71^.

In this study Silymarin supplementation to the diet reduces the liver enzymes when compared with the AF groups. The preserving effect of silymarin on livers of quail and broilers fed diet with AFB1 were reflected by the reduction of liver enzymes^56,72^.

AFB1 significantly decreased antioxidant enzymes (CAT and GSH) with higher levels of the MDA biomarkers compared to the control group^39,70,73,74^ The current results regarding the antioxidant enzymes were comparable with the majority of the recent studies. These harmful impact of AFB1 might be related to AFB1 biotransformation to intermediate metabolites of high reactivity (AFB1 8, 9 epoxide) and the release of free radicals including hydrogen peroxide (H_2_O_2_), superoxide anions (O_2_^−^), and hydroxyl radicals (^·^OH) involved in oxidative destruction^75^.

The initiation of oxidative stress and production of ROS may be a leading initiate of damaging outcomes^76^. ROS arises via the phagocytic properties of immune cells^77^. The excess of ROS in the body that surpass its capability to remove them will increase the lipid peroxidation levels in several tissues, DNA impairment and deterioration of protein expression^5,8,78^. The level of oxidative stress is based on the period of contamination, co-contamination, the synergetic effects, toxin values, animal age, species and productive phase^79^. In vitro; researches revealed that AFB1 elicited downregulation of antioxidant enzymes (SOD, GSH-Px and catalase) causing an increase of lipid peroxidation by-products (MDA) and a sharp reduction of GSH^76^.

Abou-Shehema et al.^56^ stated that 1 g silymarin/kg diet supplementation significantly improved TAC, GSH, MDA on the summer season compared with the control. Silymarin possess antioxidant properties, blocking enzymes that produce active oxygenated species and improving mitochondrial cohesiveness under stress, thereby reducing free radical damage by increasing the activity of the enzyme superoxide dismutase^53^. While the current study claims that the improvement in antioxidant indices (MDA, TAC and SOD) is attributable to silymarin, which has antioxidant activity^53^.

Studying intestinal histomorphology of rabbits is important to understand the rabbit's normal and abnormal physiological state. Normal collected histological sections from rabbits’ intestinal tract revealed an improvement in NVIS, villi breadth, length, and MTH. Recent results indicated that improved intestinal tissue structures and supplements did not cause any damage or abnormal intestinal changes. Our results agreed with Wang et al.^80^ who found that silymarin supplementation preserved the normal intestinal histology and enhanced the intestinal histomorphometry such as villi length and width. Silymarin is readily absorbed through the gastrointestinal system and achieves peak plasma concentration by 2–4 h. It has a half-life of 6–8hs in most laboratory animals with more than 80% eliminated in bile and some in urine^81^. Silymarin distention of the gastric mucosa induced by pylorus obstruction increases the vague nerve's production of acetylcholine (Ach), which acts directly on G cells and parietal cells, promoting the secretion of gastrin and histamine, respectively^82^. To determine the ideal level of silymarin for field use, further research would be necessary, especially in a practical context of rabbit production. The effective doses may vary depending on various factors such as the severity of aflatoxin contamination, the age and health of the rabbits, feed composition, and management conditions.

## Conclusion

Silymarin supplementation to weaned rabbit to ameliorate the hazard influences of aflatoxin may lead to an increase in the growth performance and ADG through enhancing villus length and mucus thickness. Moreover, silymarin is an improper supplement to reduce the oxidative stress through increased the antioxidant enzymes (SOD, CAT and TAC). Therefore, it is recommended to supplement rabbit diets’ with 30 mg Silymarin.

## Data Availability

The data is available on request. Dr.Tharwat Imbabi Tharwat.mohamed@fagr.bu.edu.eg.
